# The temporal origin of dentate granule neurons dictates their role in spatial memory

**DOI:** 10.1038/s41380-021-01276-x

**Published:** 2021-09-15

**Authors:** Nuria Masachs, Vanessa Charrier, Fanny Farrugia, Valerie Lemaire, Nicolas Blin, Wilfrid Mazier, Sophie Tronel, Marie-Françoise Montaron, Shaoyu Ge, Giovanni Marsicano, Daniela Cota, Véronique Deroche-Gamonet, Cyril Herry, Djoher Nora Abrous

**Affiliations:** 1 Univ. Bordeaux, INSERM, Neurocenter Magendie, Neurogenesis and Pathophysiology Group, U1215, F-33000 Bordeaux, France; 2 Univ. Bordeaux, INSERM, Neurocenter Magendie, Energy Balance and Obesity Group, U1215, F-33000 Bordeaux, France; 3 Program in Neuroscience, SUNY at Stony Brook, Stony Brook, New York, NY USA; 4 Univ. Bordeaux, INSERM, Neurocenter Magendie, Endocannabinoids and Neuroadaptation Group, U1215, F-33000 Bordeaux, France; 5 Univ. Bordeaux, INSERM, Neurocenter Magendie, Psychobiology of Drug Addiction Group, U1215, F-33000 Bordeaux, France; 6 Univ. Bordeaux, INSERM, Neurocenter Magendie, Neuronal Circuits of Associative Learning Group, U1215, F-33000 Bordeaux, France

**Keywords:** Neuroscience, Psychiatric disorders

## Abstract

The dentate gyrus is one of the only brain regions that continues its development after birth in rodents. Adolescence is a very sensitive period during which cognitive competences are programmed. We investigated the role of dentate granule neurons (DGNs) born during adolescence in spatial memory and compared them with those generated earlier in life (in embryos or neonates) or during adulthood by combining functional imaging, retroviral and optogenetic tools to tag and silence DGNs. By imaging DGNs expressing Zif268, a proxy for neuronal activity, we found that neurons generated in adolescent rats (and not embryos or neonates) are transiently involved in spatial memory processing. In contrast, adult-generated DGNs are recruited at a later time point when animals are older. A causal relationship between the temporal origin of DGNs and spatial memory was confirmed by silencing DGNs in behaving animals. Our results demonstrate that the emergence of spatial memory depends on neurons born during adolescence, a function later assumed by neurons generated during adulthood.

## Introduction

Adolescence is a critical period during which the brain undergoes a series of structural and functional maturation steps allowing the transition from childhood to adulthood [1, 2]. Ranging from approximately postnatal weeks 3–4 to 6–7 in rodents, it has been suggested that this period is a stage in which neural circuitry and synapses are sculpted to support the emergence of social behaviour [2] and the transition from simple to complex forms of learning [3, 4]. This period is also often associated with heightened emotional reactivity, increased impulsivity and vulnerabilities to psychological disorders [1, 2]. Adolescent brain neuronal development in particular has been studied in this context. The maturation of the prefrontal cortex (and its connections) has been linked to pruning, perineuronal net maturation, myelination and neurochemical changes (excitatory/inhibitory balance, endocannobinoid systems, dopaminergic sprouting) to name but a few [5, 6]. In contrast, relatively little is known about the neurobiological developmental changes occurring during adolescence that may be involved in the maturation of spatial memory function.

The dentate gyrus (DG) of the hippocampus is a heterogeneous structure composed of different populations of dentate granule neurons (DGNs), most of which are generated during the early postnatal period [7] but also throughout adult life in a process called adult neurogenesis [8, 9]. In recent years, some investigations have focused on the study of morphological and functional differences between adult and developmental DGNs showing that these populations present major morphological differences [10, 11], and are recruited in different learning processes [12, 13]. In addition, adult-born DGNs (Adu-DGNs) undergo a process of dendritic plasticity in response to learning [14–16] whereas developmental born neurons seem to be insensitive [14, 15].

However, the specific contribution of DGNs born during adolescence (here called Adol-DGNs) to hippocampal-dependent behaviour is largely unknown. There are a few studies in which Adol-DGNs were indirectly manipulated using either stress [17, 18], running [19], social isolation [20] or genetic mutations [21, 22]. All of these manipulations lead to behavioural consequences on emotion and learning in adult animals, but their indirect nature did not allow a firm causal relationship between Adol-DGNs and behaviour to be established. Thus, new methods that specifically target Adol-DGNs are needed in order to directly assess their involvement in learning and memory, and to establish whether their role differs from those generated earlier (in embryos or neonates) or at adulthood.

In this study we investigated whether the temporal origin of DGNs dictates their role in spatial memory by combining functional imaging, retroviral and optogenetic approaches. Using Zif268 [12] an immediate early gene (IEG), critical for the stabilisation of long-lasting memories [23], we found that DGNs generated in adolescent rats (and not embryos or neonates) are transiently involved in memory processing during early adulthood whereas DGNs generated in adulthood are not recruited until a later time point when animals are older. The causal relationship between the temporal origin of DGNs and spatial memory was then demonstrated using retroviral and optogenetic approaches to birth date and silence DGNs during learning [24]. Our results demonstrate for the first time a functional specificity of developmental and adult neurogenesis in hippocampal spatial memory.

## Materials and methods

### General methods

#### Animals

A total number of 292 male and 21 pregnant female Sprague-Dawley rats (OFA, Janvier, France) were used to perform these experiments (detailed in Tables 1 and 2). Rats were housed under a 12 h/12 h light/dark cycle (light off at 8 p.m.) with ad libitum access to food and water. Temperature (22 °C) and humidity (60%) were kept constant. Experimental procedures were carried out following Directive 2010/63/EU of the European Parliament and of the Council of 22 September 2010. Animal studies were approved by the ethical committee of the University of Bordeaux (CEEA50, Dir 13105, #5306).

**Table 1:** Summary of the experimental groups and experiments for IEGs experiments.

Experiment	Animal’s age at the time of Xdu injection	DGNs’ age at the time of WM training	Group size	XdU (mg/kg)
1. IEGs activation of neurons Adol-1M (Fig. 1A–D)	P28	1 month	Control = 9; Learning = 11; Swim = 9; Cued = 9	IdU: 1 × 150
2. IEGs activation of neurons Adol-2M (Fig. 1E–H)	P28	2 months	Control = 5; Learning = 9; Cued = 5	IdU: 1 × 100
3. IEGs activation of neurons Adol-3M (Fig. 1I–L)	P28	3 months	Control = 5; Learning = 6 Cued = 6	CldU: 1 × 50
4. IEGs activation of neurons Adu-1M (Fig. 2A, B)	2 M	1 month	Control = 3 Learning = 6	BrdU: 3 × 100
5. IEGs activation of neurons Adu-2M (Fig. 2A, B)	2 M	2 months	Control = 3 Learning = 5	BrdU: 3 × 100
6. IEGs activation of neurons Adu-3M (Fig. 2A, B)	2 M	3 months	Control = 5 Learning = 7	BrdU: 3 × 100
7. IEGs activation of neurons generated during embryonic and neonatal periods (Fig. 2C–E)	E18.5 P14	10 weeks 12 weeks	Control = 6 Learning = 13 Cued = 8	CldU: 2 × 50 IdU: 1 × 50﻿

**Table 2:** Summary of the experimental groups and experiments for optogenetics experiments.

Experiment	Animal’s age at the time of Arch injection	DGNs’ age at the time of WM training	Group size
1. Optogenetic inhibition of Adol-1M DGNs (Fig. 3A–E)	P28	1 month	**Adol**-DGNs-Arch-No-Light = 14 **Adol**-DGNs-Arch-Light = 6 **Adol**-DGNs-GFP-Light = 12
2. Optogenetic inhibition of Adol-2M DGNs (Fig. 3F–J)	P28	2 months	**Adol**-DGNs-Arch-No-Light = 13 **Adol**-DGNs-Arch-Light =13 **Adol**-DGNs-GFP-Light =13
3. Optogenetic inhibition of Adol-4M DGNs (Fig. 3K–O)	P28	4 months	**Adol**-DGNs-Arch-No-Light =10 **Adol**-DGNs-Arch-Light = 8 **Adol**-DGNs-GFP-Light = 9
4. Optogenetic inhibition of Adu-1M DGNs (Fig. 4A–D)	2 M	1 month	**Adu**-DGNs-Arch-No-Light = 10 **Adu**-DGNs-Arch-Light = 11
5. Optogenetic inhibition Adu-6M DGNs (Fig. 4E–H)	2 M	6 months	**Adu**-DGNs-Arch-No-Light = 11 **Adu**-DGNs-Arch-Light = 12
6. Optogenetic inhibition of Neo-DGNs (Fig. S4)	P3	2 months	**Neo**-DGNs Arch-No-Light = 7 **Neo-**DGNs Arch-Light = 13

#### Injection of thymidine analogues

BrdU (5-Bormo-2′-deoxyuridine), IdU (5-Iodo-2′-deoxyuridine) and CldU (5-chloro-2′-deoxyuridine) were dissolved in Phosphate Buffer (pH 8.4), 1 N NH4OH/NaCl and NaCl, respectively. CldU and IdU injections were given with equimolar doses of BrdU (50, 100 and 150 mg/kg, Table 1) [12, 25].

#### Water maze training

The apparatus consisted of a circular plastic swimming pool (180 cm diameter, 60 cm height) in a 330 cm × 300 cm room that was filled with water (20 ± 1 °C) rendered opaque by the addition of a white cosmetic adjuvant. Before training, the animals were habituated to the pool for 1 min per day for 2 days. During training, the Learning group (L) was composed of animals that were required to locate a submerged platform, which was hidden 1.5 cm under the surface of the water in a fixed location, using the spatial cues available within the room (according to a previously described reference memory protocol [25]). All rats were trained for four trials per day (90 s with an intertrial interval of 30 s and released from three starting points used in a pseudorandom sequence each day). If an animal failed to locate the platform, it was placed on that platform at the end of the trial. The time taken to reach the platform was recorded using a video camera that was secured to the ceiling of the room and connected to a computerised tracking system (Videotrack, Viewpoint). In the first 3 experiments, three control groups were used. The home cage group (HC) consisted of animals that were transferred to the testing room at the same time and with the same procedures as the Learning group but that were not exposed to the water maze. The Swim group (S) was a Yoked control group for the stress and motor activity associated with the water maze training. The Swim group was composed of rats that were placed in the pool without the platform and were paired for the duration of the trial with the Learning animals. The third control group was the Cued group (C) which was composed of rats that were trained to find a visible platform in a fixed location. Animals in this group were tested for four trials per day (90 s with an intertrial interval of 30 s and beginning from three different starting points used in a pseudorandom sequence). Trained animals and different age-matched control groups were perfused transcardially 90 min after the last trial.

#### Retrovirus production

The murine Moloney leukaemia virus-based retroviral vector CAG-GFP has been described previously [14] and is a gift from Prof. F. H. Gage (Salk Institute, La Jolla, CA). The retrovirus expressing an inhibitory optogene *Archaerhodopsin*-3 (Arch-EGFP) was generated by Dr S. Ge [24]. High titres of retroviruses (between 5 × 10^8^ and 5 × 10^9^) were prepared with a human 293-derived retroviral packaging cell line (293GPG) [26] kindly provided by Dr Dieter Chichung Lie (Friedrich-Alexander Universität Erlangen-Nürnberg, Erlangen, Germany). Virus-containing supernatant was harvested three days after transfection with Lipofectamine 2000 (Thermofisher). This supernatant was then cleared from cell debris by centrifugation at 3500 rpm for 15 min and filtered through a 0.45 µm filter (Millipore). Viruses were concentrated by two rounds of centrifugation (19,500 rpm 2 h) and resuspended in PBS.

#### Stereotaxic injections of retrovirus

The retroviral solution was injected bilaterally into the dorsal area of the septal DG. Adult and adolescent rats were anaesthetised in a box using a mix of oxygen and 3% isoflurane and maintained asleep with 2% isoflurane for the duration of surgery in a stereotaxic apparatus. P3 rats were anaesthetised by placing them in ice. Animals also received lidocaine (0.1 ml, sub-cutaneous, Vetoquinol) at the incision site and metacam (1 ml/kg, sub-cutaneously, Boehringer Ingelheim). Glass micropipettes connected to a Hamilton syringe placed into a micro injector (KDS legato 130) directly attached to the stereotaxic apparatus were used. Injections were administered using the following coordinates [27]: For Neo-DGNs groups, [AP]: −1.2 mm; lateral [L]: ±1.35 mm; [P]: −2.6 mm; Adol-DGNs groups, [AP]: −2.8 mm; [L]:±1.5 mm; [P]: −3.5 mm and for Adu-DGNs groups, [AP]: −3.3 mm; [L]: ±1.6 mm; deepness [P]: −4.2 mm. 1 (Neo-DGNs), 2 (Adol-DGNs) or 3 µl (Adu-DGNs) of viral suspension were injected at a flow rate 0.25 L/min. Cannulas were maintained in position for 3 min after the end of the injections to let the suspension infuse. The skin was then stitched with absorbable sutures and the rat was placed in a recovery chamber (37 °C) until it woke up.

#### Electrophysiological recording

Animals were deeply anaesthetised (Xylazine 16.7 mg/kg plus Ketamine 167 mg/kg, intraperitoneal, Bayer santé and Mérial, Germany, respectively) and sacrificed. Dissected brain was immediately immerged in ice-cold oxygenated cutting solution (in mM: 180 Sucrose, 26 NaHCO_3_, 11 Glucose, 2.5 KCl, 1.25 NaH_2_PO_4_, 12 MgSO_4_, 0.2 CaCl_2_, saturated with 95% O2–5% CO_2_). 350 mm slices were obtained using a vibratome (VT1200S Leica, Germany) and transferred to a 34 °C bath of oxygenated aCSF (in mM: 123 NaCl, 26 NaHCO3, 11 Glucose, 2.5 KCl, 1.25 NaH_2_PO_4_, 1.3 MgSO_4_, 2.5 CaCl_2_; osmolarity 310 mOsm/l, pH 7.4) for 30 min and then cooled down progressively to room temperature (RT; 23–25 °C) in oxygenated aCSF. After a 45 min recovery period at RT, slices were anchored with platinum wire at the bottom of the recording chamber and continuously bathed in oxygenated aCSF (RT; 2 ml/min) during recording.

Fluorescent DGN cells were identified using fluorescence/infra-red light (pE-2 CoolLED excitation system, UK). Neuron action potential firing was monitored in whole-cell current-clamp recording configuration. Patch electrodes were pulled (micropipette puller P-97, Sutter instrument, USA) from borosilicate glass (O.D. 1.5 mm, I.D. 0.86 mm, Sutter Instrument) to a resistance of 2–4 mΩ. The pipette internal solution contained [in mM: 125 potassium gluconate, 5 KCl, 10 Hepes, 0.6 EGTA, 2 MgCl2, 7 Phosphocreatine, 3 adenosine-5′-triphosphate (magnesium salt), 0.3 guanosine-5′-triphosphate (sodium salt) (pH adjusted to 7.25 with KOH; osmolarity 300 mOsm/l adjusted with d-Mannitol)] with added biocytin 0.4% (liquid junction potential −14.8 mV was corrected on the data and statistics). Electrophysiological data were recorded using a Multiclamp 700B amplifier (Molecular devices, UK), low-pass filtered at 4 kHz and digitised at 10 Hz (current clamp) or 4 Hz (voltage clamp) (Digidata 1440A, Molecular devices, UK). Activation of Arch was obtained after illumination of the recorded area at 570 nm (pE-2 CoolLED excitation system, UK). Signals were analysed offline (Clampfit software, pClamp 10, Molecular devices, UK).

#### Optic fibre implantation

Home-made optic fibre implants were constructed following a protocol previously described [28]. The implantation step was carried out during a second surgical procedure two weeks before the behaviour, following the same protocol as that described for the retroviral injection. The skull was scratched and three anchor screws were fixed (one in the anterior area and two in the posterior area). The two optic fibres (length: 4.5 mm, diameter: 200 µm, numeric aperture (NA: 0.37)) were lowered with a speed of ~2 mm/min upwards to the RV injection site with the following stereotaxic coordinates [27]: Adu-1M group: [AP]: −3.3 mm; [L]: ±1.6 mm; [P]: −4 mm from skull surface; Adu-6M group: [AP]: −3.7 mm; [L]: ±1.6 mm; [P]: −4 mm from skull surface; Adol-1M group: [AP]: −3.2 mm; [L]: ±1.5 mm; [P]: −3.6 mm from bregma; Adol-2M and Adol-4M groups: [AP]: −3.4 mm; [L]: ±1.5 mm; [P]: −3.8 mm; Neo-DGNs group: [AP]: −3.3 mm; [L]: ±1.6 mm; [P]: −4 mm (see below for the group description). Fibres were stabilised using dental cement. When the cement was dry, scalps were sutured and disinfected with local antiseptic treatment (Betadine). All rats were followed up until the start of the behaviour. During handling, fur was checked and the scar disinfected systematically. Rats were handled every day to habituate them before the behavioural procedure.

#### Optogenetic manipulation of DGNs during training in the water maze

The water maze (a circular pool with a diameter of 1.80 m and 60 cm deep) was placed in a room (375 cm × 370 cm) equipped with a green laser source (OptoDuet 532,5 nm, 200 mW, Ikecool) that was connected to a rotatory joint to avoid tangling of the patch cords connected to the intra-dentate optic fibres. Light intensity at the optic fibre cable tip was checked every day before the beginning of the session (8–10 mV). Illumination was applied throughout the session at 1 Hz pulses of 750 ms. The same protocol as that described in the section ‘Water Maze training’ was used in this training. The time taken to reach the platform was recorded using a video camera connected to a computerised tracking system (Polyfiles, Imetronic). When animals reached the plateau phase, training was stopped and 72 h later animals were tested in finding the platform. During the probe test, the hidden platform was removed and the time spent in each quadrant was quantified.

#### Immunohistochemistry

Animals were perfused transcardially with a phosphate-buffered 4% paraformaldehyde solution. After 1 week of fixation, brains were cut using a vibratome as previously described [25]. Free-floating sections (50 µm) were processed using a standard immunohistochemical procedure to visualise the thymidine analogues (BrdU, CldU, IdU) and retrovirus-expressing EGFP in alternating one-in-ten sections using different anti-BrdU antibodies from different vendors (for BrdU: mouse primary at 1/100, Dako; CldU: rat primary at 1/1000, Accurate Chemical; IdU: mouse primary at 1/1000, BD Biosciences) and with a rabbit anti GFP (Millipore, 1/2000). Bound antibodies were visualised with horse anti-mouse (1/200, Abcys for BrdU and Idu), goat anti-rat (1/200, Vector for CldU) and goat anti-rabbit (1/200, Vector for GFP). The number of XdU-immunoreactive (IR) cells in the granule and subgranular layers (GL) of the DG was estimated with systematic random sampling of every tenth section along the septo-temporal axis of the hippocampal formation using a modified version of the optical fractionator method. Indeed, all of the X-IR cells were counted on each section and the resulting numbers were tallied and multiplied by the inverse of the section sampling fraction (1/ssf = 10). This counting was performed blind by the person who carried it out.

The activation of embryonic, neonatal, adolescent and adult generated neurons was examined using immunohistofluorescence. To visualise the cells that incorporated thymidine analogues, one section out of ten was incubated with BrdU antibodies (BrdU, 1/1000, CldU, 1/500, Accurate Chemical; IdU, 1/500, BD Bioscience). Bound antibodies were visualised with Cy3-goat anti-rat antibodies (1/1000, Jackson for CldU and BrdU) or Cy3-goat anti-mouse antibodies (1/1000, Jackson for IdU). Sections were also incubated with rabbit anti-Zif268 (1/500, Santa Cruz Biotechnology). Bound antibodies were visualised with Alexa488-goat anti-rabbit antibodies (1/1000, Invitrogen). Primary antibodies for CldU (or IdU or BrdU) and IEG (Zif268) were incubated simultaneously at 4 °C for 72 h, and secondary antibodies were incubated simultaneously at RT for 2 h. CldU-IEG and IdU-IEG labelling were not analysed on the same sections, given the cross-reactivity between the secondary (and not primary) antibodies [12]. The sections were then incubated with the two secondary antibodies. The phenotype of the CldU-IR cells was examined by immunofluorescence labelling using NeuN (1:1000; Millipore) revealed with a Cy5-goat anti-mouse (1:1000; Jackson) and the phenotype of IdU-IR cells using Calbindin (Calb, 1:250; Santa Cruz Biotechnologies) revealed with Alexa-Fluor 647 donkey anti-goat (1/1000; Jackson). Double labelling was determined using a SPE confocal system with a plane apochromatic 63X oil lens (numerical aperture 1.4; Leica). The percentage of XdU cells expressing IEG and the number of XdU-cells expressing NeuN or Calb were calculated as follows: (Nb of XdU^+^/IEG + cells)/[(Nb of XdU^+^/IEG^-^ cells) + (Nb of XdU^+^/IEG + cells)] × 100. All sections were optically sliced in the Z plane using a 1 µm interval and cells were rotated in orthogonal planes to verify double labelling. In all experiments, a minimum of 200 developmentally generated neurons were analysed per rat.

The morphometric analysis of virus-labelled neurons was performed with an ×100 objective as previously described [10]. Measurements of dendritic parameters as well as Sholl analysis were performed with the Neurolucida (software Microbrightfield, Colechester, VT, USA). Only neurons from the suprapyramidal layer were analysed. The analysis of the dendritic arborisation was performed blind by the person who carried it out. Moreover, only neurons that reached the edge of the molecular layer and had at least two ramification points, were analysed. Data shown is from at least five rats per group with a minimum of four neurons per rat (Supplementary Table 3).

### General procedures

#### Immediate early genes experiments (Table 1)

In the first three functional imaging experiments, we ascertained whether neurons born during the adolescent period were activated by spatial learning. Animals were delivered at P21 days old and they were kept in collective cages (2–3 rats per cage). One week later, rats received one injection of IdU or CldU when 28 days-old. One week before the beginning of the water maze training, animals were housed individually. These animals were randomly distributed into 4 (Home Cage, Swim, Cued and Learning) or 3 (Home Cage, Cued and Learning) experimental groups and were trained in the water maze (see above) 1, 2 or 3 months after the XdU injections.

In experiments four, five and six, we examined whether neurons born during adulthood (Adu-DGN) were activated by spatial learning. Animals were delivered at 6 weeks old and received three injections of BrdU when 2 months old. They were housed in collective cages (2–3 rats per cage) until 1 week before the behavioural training when they were transferred to individual cages. These animals were randomly distributed into 2 experimental groups (Home Cage and Learning) and were trained in the water maze one, two or three months after the XdU injections.

In the seventh experiment, we examined whether neurons born during the embryonic period and those born during the second postnatal week (P14) in the same rats were activated by spatial learning. Pregnant female (*n* = 12, 3 months of age on delivery) rats were individually housed in transparent cages and the mothers received two injections of CldU at E18.5 and E19.5. At 14 days-old (P14), males received one injection of IdU. The litters were raised by their biological mothers until weaning (21 days after birth). After weaning, only the male progeny were kept in collective cages (2–3 rats per cage) and transferred to individual cages 1 week before the beginning of the behavioural training. The animals were randomly distributed into three experimental groups (Home Cage, Cued and Learning) and trained in the water maze when they were 3 months-old.

#### Optogenetic experiments (Table 2)

In the first three optogenetic experiments, we wanted to see the effect of inhibiting DGNs born during adolescence on spatial learning (Adol-DGNs). 135 males were delivered at P21 days old and kept in collective cages (2–3 rats per cage). One week later, P28 day-old animals were bilaterally injected with retrovirus (expressing either Arch or GFP) in the DG. Two weeks before the water maze training, optic fibres were implanted bilaterally in the DG and the animals were moved afterwards to individual cages. Rats were trained in the water maze 1 (Adol-1M), 2 (Adol-2M) or 4 months (Adol-4M) after tagging the cells.

Experiments four and five were performed to assess the effect of silencing adult-born DGNs on spatial learning (Adu-DGNs). 60 males were delivered at the age of 6 weeks and held in collective cages (2–3 rats per cage). At 8 weeks old, animals were bilaterally injected with retrovirus (expressing Arch) in the DG, and as in previous experiments, 2 weeks before the water maze training, optic fibres were placed in the DG and rats were transferred to individual cages. Rats were trained 1(Adu-1M) or 6 months (Adu-6M) after the viral injection.

Finally, for the neonate DGN experiment (Neo-DGNs), 43 male pups from 9 different females were bilaterally injected with retrovirus (expressing Arch). They were raised by their biological mothers until weaning, and they were kept in collective cages (2–3 rats per cage) before optic fibres were implanted 2 weeks before water maze training which was performed 2 months after the retroviral injection. Animals were randomly assigned in the two groups.

At the end of all of these experiments, only animals with GFP-labelled-cells in both hemispheres and the fibre optic implants at the correct site (at the same anteroposterior levels of GFP-DGNs, see Supplementary Fig. 1) were considered for further analysis (Adu-DGN experiments: *n* = 44, Adol-DGN experiments: 64; Neo-DGNs experiment: *n* = 20, for more details see Table 2).

#### Statistical analysis

All statistical analyses were performed using Statistica software (Statsoft) and results are reported in Supplementary Tables 1–3. One-way ANOVA with between factors (‘Group’, ‘Light’) or a within factor (‘Quadrant’ factor) were used as well as two-way ANOVAs with a between factor (‘Group’, ‘Light’) and within factors (‘Days’, ‘Distance to the soma’). ANOVA were followed by Newman-Keuls post-hoc analysis when appropriate. In the case of comparison of two independent groups or comparison to chance levels, Student *t* tests were used. The relationship between memory retention and dendritic morphology was evaluated using a Spearman correlation test. For all tests, significance was set at *p* = 0.05. All data are presented as mean ± s.e.m.

## Results

### The temporal origin of DGNs determines their activation during spatial learning

**Fig. 1:**
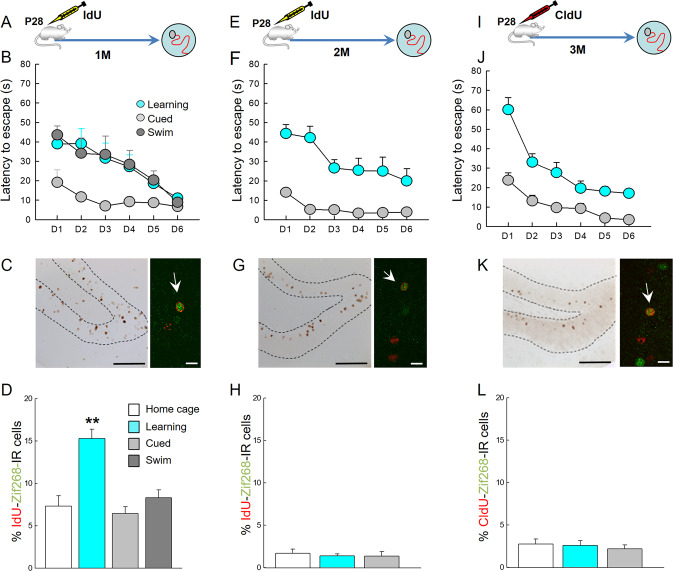
DGNs born in adolescents are recruited by spatial learning within a defined temporal window. **A**, **E**, **I** Experimental designs. **B**, **F**, **J** Learning curves. **C**, **G**, **K** Left panel: illustration of Idu- or CldU- labelled cells (Bar Scale = 80 µm). Right panel: confocal example of Idu-Zif-268- or CldU-Zif-268- labelled cells (arrows indicate double labelled cells; Bar Scale = 10 µm). Spatial learning increased the number of activated Adol-DGNs when the cells were 1 month old (**D**) and not when they were 2 month-old (**H**) or 3 month-old (**L**). **p ≤ 0.01 compared to other groups.

Adol-DGNs born in P28 male rats were labelled using thymidine analogues IdU (5-Iodo-2′-deoxyuridine) or CldU (5-chloro-2’-deoxyuridine) [12] (Table 1) and their recruitment by training in the water maze (WM) was examined by quantifying in IdU or CldU-labelled cells, the expression of Zif268, a proxy for neuronal activity [12, 29]. In the first experiment, rats were trained one month after IdU injection (Fig. 1A,B). IdU-labelled cells differentiated into neurons, as revealed by co-expression with the adult neuronal marker NeuN (% of IdU expressing NeuN, Home Cage: 97.92 ± 0.66%, Learning: 96.57 ± 0.82%; *t*_43_ = 1.28, *p* = 0.21) and were located in the inner granule cell layer. The percentage of activated Adol-DGNs (IdU-Zif268-IR cells) in the Learning group was superior to that measured in the age-matched Home Cage control group (Fig. 1D). Learning to find a visible platform (Cued learning), a hippocampal-independent task, or stress-physical activity (Swim group) did not activate Adol-DGNs (Supplementary Table 1 for statistical analysis) demonstrating that Adol-DGN activation was specific to learning. We then investigated whether the age of these Adol-DGNs at the time of training was critical or whether their contribution was stable over time. We tagged Adol-DGNs with IdU or CldU and trained rats in the WM, two or three months later (Fig. 1E,F,I,J). Under these conditions (2 or 3 month-old neurons), Adol-DGNs were not activated by training (Fig. 1H, L) indicating that Adol-DGNs were activated by learning within a defined temporal window.

**Fig. 2:**
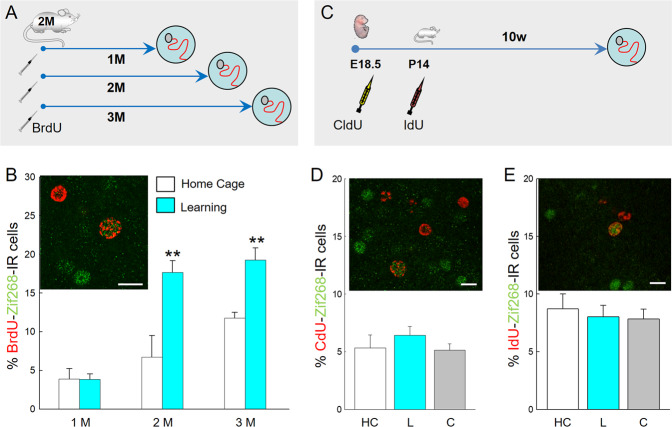
Mature Adu-DGNs are activated by learning in contrast to DGNs born in embryos or neonates. **A** Experimental design: DGNs born in 2-month-old rats were tagged with BrdU. Rats were tested 1, 2 or 3 months after the injections. **B** Illustration and quantitative analysis of the percentage of BrdU cells expressing Zif-268. **C** Experimental design: DGNs born in embryos were tagged with CldU (injected into the pregnant mothers) and DGNs in the same animals born at P14 were labelled with IdU. Ten weeks after the last injection animals were trained in the water maze. **D** Illustration and quantification of the percent of CldU cells (in red) expressing Zif268 (in green). **E** Illustration and quantitative analysis of the percent of IdU cells expressing Zif268. HC: home Cage; L: Learning, C: Cued. Arrows indicate double labelled cells. **p < 0.01 in comparison to the HC. (Bar Scale = 10 µm).

Then we investigated whether the recruitment of Adol-DGNs was specific to this neuronal population by first focusing on neurons born during adulthood (Adu-DGNs) (Fig. 2A). To this end, 2-month-old rats were injected with BrdU (5-Bormo-2′-deoxyuridine) and submitted to training one month later. We found that in this condition, the percentage of BrdU-Zif268-IR neurons was not influenced by training (Fig. 2B and Supplementary Table 1). In contrast, when the BrdU pulses and training interval were increased to 2 or 3 months, Adu-DGNs were activated by learning (Fig. 2B and Supplementary Table 1). Next, we analysed neurons generated during embryogenesis and during the neonatal period to determine whether this activation was specific to the neurons born during either adolescence or adulthood. Neither 3-month-old DGNs born during either embryogenesis (Emb-DGNs) or the neonatal period (Neo-DGNs) were activated by spatial learning in adult rats (Fig. 2C–E and Supplementary Table 1). Taken together, these data suggest that: i) Adol-DGNs are recruited by learning only when they are one month-old ii) Adu-DGNs (and not neurons born during development) are recruited after the age of 2 months.

### Inhibition of Adol- and Adu-DGNs impairs the expression of memory in a time-dependent manner

**Fig. 3:**
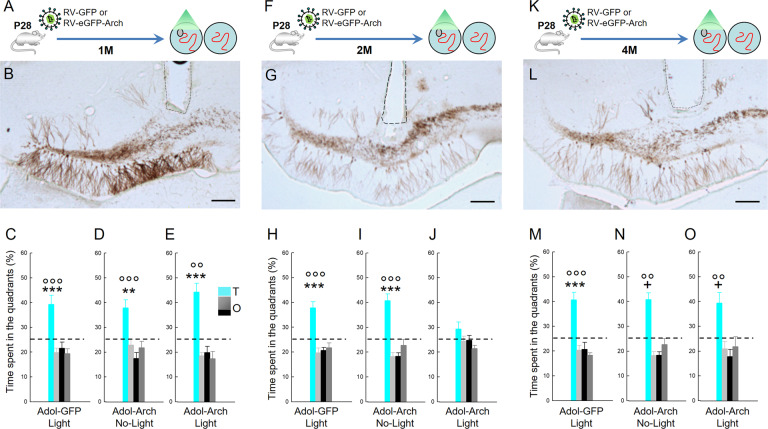
Optical silencing of Adol-DGNs transiently affects memory retrieval. **A**, **F**, **K** Experimental designs. Illustration of Adol-DGNs **B**, **G**, **L** infected with RV-GFP or RV-eGFP-Arch; dashed line, position where optic fibres were placed (Bar Scale = 200 µm). Silencing **C**–**E** 1-month old Adol-DGNs during training did not impair memory retention, whereas **H**–**J** silencing 2-month-old Adol-DGNs impaired the ability of the animals to remember the platform location. When 4 months old, silencing Adol-DGNs did not influence memory retention **M**–**O** ^+^at least at *p* < 0.05 and ^**^p < 0.01. ^***^p < 0.001 compared to the other quadrants. °°*p* ≤ 0.01 and °°°*p* ≤ 0.001 compared to chance level (25%). T: target quadrant. O: other quadrants.

To confirm the contribution of Adol-DGNs and Adu-DGNs in spatial learning during different time windows, we used an optogenetic approach to silence DGNs using a retrovirus expressing eGFP-tagged *Archaeorhodopsin* (RV-Arch-EGFP), a tool that allows us to inhibit the cells expressing Arch in presence of light [24]. We first validated this tool in the rat DGNs by showing that optical inhibition reversibly silenced RV-Arch-EGFP-expressing neurons in a power-dependent manner as previously reported in mice [24] (Supplementary Fig. 2). Then, we bilaterally injected the RV-Arch-EGFP into the DG of 28 days-old animals, and rats were trained in the WM 1, 2 or 4 months after tagging the cells. Two weeks before training, optic fibres were implanted bilaterally above the DG and half of the animals were trained with the light on (Adol-DGNs-Arch-Light) and the other half with the light off (Adol-DGNs-Arch-No-Light). No differences were observed between groups during the learning phase (Supplementary Fig. 3A–C). Seventy-two hours later, memory retention was measured in a probe test. During this test, the platform was removed and the optic fibres connected, but with no light. When there was a lapse of 1 month between retroviral labelling and behavioural testing, both Adol-DGNs-Arch-No-light and Adol-DGNs-Arch-light animals remembered the platform position as they spent more time in the quadrant previously associated with the platform (Target quadrant, T) (Fig. 3D,E and Supplementary Table 2 for complete statistical analysis) compared to the other quadrants (O). In addition, the time spent in the target quadrant differed significantly from the chance level (>25%). In subsequent experiments, rats were trained in the WM 2 or 4 months after tagging the cells. In contrast, retrieval was impaired in the Adol-DGNs-Arch-light group when there was a 2-month delay between retroviral labelling and the behavioural test (Fig. 3I,J and Supplementary Table 2 for complete statistical analysis). Interestingly, no alteration was observed when the delay was increased to 4 months (Fig. 3N, O and Supplementary Table 2 for complete statistical analysis). To confirm that the effect of the light was not responsible for the impairment observed, an extra group of animals was injected bilaterally with RV-GFP without expressing *Archaeorhodopsin* and these animals were tested at the same time as the previous ones. No light effect was found for any analysed time points, either in learning (Supplementary Fig. 3A–C) or in retrieval (Fig. 3C, H, M). After this verification, only the Arch-No-Light group was used as a control in the following experiments.

To ascertain that the contribution of Adol-DGNs was due to the date of birth (Adolescence) and not the age of the population under study (2 months), RV-Arch-EGFP was injected bilaterally into the DG of 3-day-old animals that were trained 2 months later. Both Neo-DGNs-Arch-No-Light and Neo-DGNs-Arch-Light learned and remembered the task (Supplementary Fig. 4 and Supplementary Table 2).

**Fig. 4:**
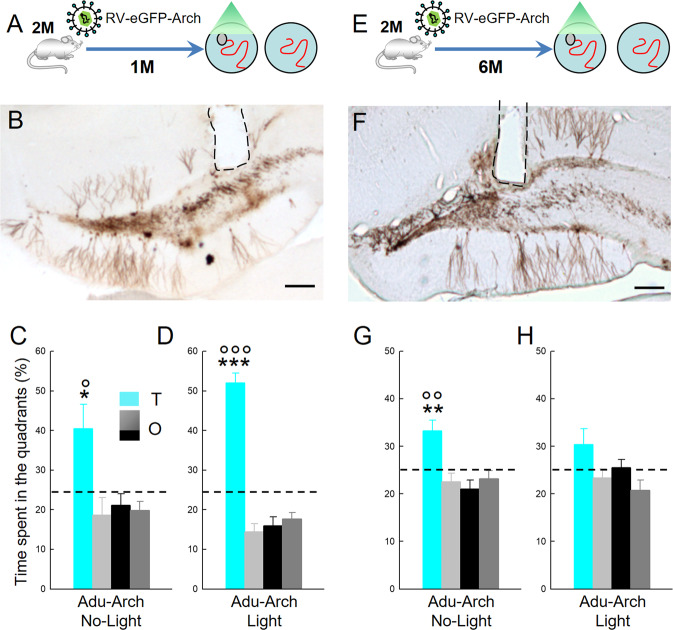
Optical inhibition of mature Adu-DGNs affects memory retrieval. **A**, **E** Experimental designs. Illustration of Adu-DGNs **B**, **F** infected with RV-Arch-EGFP; dashed line, position where the optic fibres were placed (Bar Scale = 200 µm). Silencing **C**, **D** 1-month-old Adu-DGNs during training did not impair memory retention, whereas **G**, **H** silencing 6-month-old Adu-DGNs impaired the ability of the animals to remember the platform location. + at least at *p* < 0.05 compared to other quadrants. **p ≤ 0.01 ***p ≤ 0.001 compared to other quadrants. °°*p* ≤ 0.01 and °°°*p* ≤ 0.001 compared to chance level (25%). T: target quadrant. O: other quadrants.

Then we focused our attention on Adu-DGNs by injecting RV-Arch-EGFP bilaterally in 2-month-old animals and testing the impact of silencing 1- and 6-month-old Adu-DGNs. A one month interval was chosen to confirm that the effect observed when manipulating Adol-DGNs (Fig. 3I,J) was specific to this wave of DGNs and not the age of the rat. The 6-month time point was chosen based on our previous experiments showing that Adu-DGNs are recruited by learning even when these neurons are 6 months old [12]. In both experiments, half of the animals were trained with the light on (Adu-DGNs-Arch-Light) and the other half with the light off (Adu-DGNs-Arch-No-Light). No differences were observed between groups during learning (Supplementary Fig. 3D, E). We then performed the probe test and we observed that in the Adu-1M, both Adu-DGNs-Arch-No-light and Adu-DGNs-Arch-light rats remembered the platform position since they spent more time in it compared to the other quadrants (Fig. 4C,D); in addition the time spent in the target quadrant was significantly different from the chance level (>25%). When a delay between the retroviral labelling and behavioural testing was increase to 6 months (Adu-6M), we observed that Adu-DGNs-Arch-No Light (8-month-old animals) had weaker memories compared with younger control groups from the other experiments (Animals between two and four-months of age, see performance in Fig.3C, H, M; Fig.4C; and Supp Fig.4). Even though, they could still remember the target quadrant where the platform was (Fig. 4G and Supplementary Table 2 for complete statistical analysis). In contrast, Adu-DGNs-Arch-Light animals were unable to remember the platform position as revealed by the time spent in the target quadrant which was no different to that spent in the other quadrants or to the chance level (Fig. 4G, H and Supplementary Table 2 for complete statistical analysis). These data indicate that, as animals become older, memory retention depends on mature adult-born neurons.

### Different morphological features of dendritic arborisation among DGN populations

**Fig. 5:**
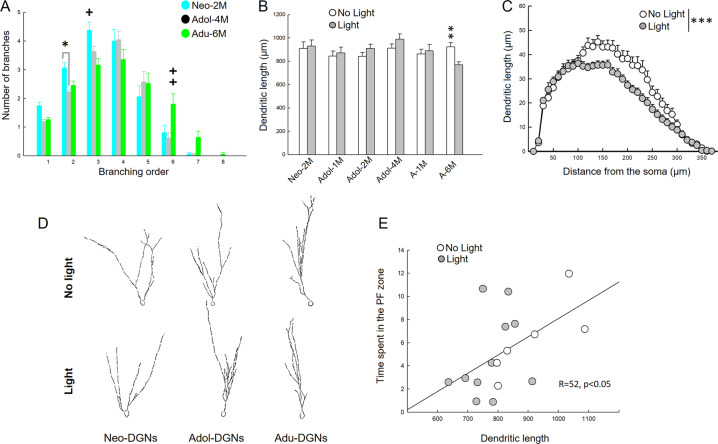
Different populations of DGNs show distinct features in branching arborisation and they are distinctly modified by the light. **A** Dendritic branching shows diverse profiles of different mature DGN populations (interaction group x order: *F*_14,735_ = 3.630, *p* < 0.0001). Neo-DGNs show an increase in low order dendrites, while the Adu-DGNs show an increase in higher order dendrites. When the effect of light on the dendritic arbour was examined, a reduction in the total length was only observed in Adu-DGNs, an effect (**B**) associated with a decrease in dendritic complexity (**C**) (See also, Supplementary Table 3). **D** Representative tracings of the DGN populations analysed. Correlation between dendritic length and the time spend in the PF zone (**E**) during the probe test of the Adu-DGNs 6 M (ddl = 17, r = 52, p < 0.05). *p ≤ 0.05, **p ≤ 0.01, ***p ≤ 0.001, + at least at *p* < 0.05 + + at least at *p* ≤ 0.01.

Finally, the morphology of the dendritic arbour of DGNs born at different ontogenetic stages and the effect of light on their organisation were analysed. Clearly, Neo-DGNs exhibited specific features compared to the other cohorts such as multiple primary dendrites, with a wider branching angle and a more complex organisation of the proximal part of the dendritic arbour (Fig. 5A and Supplementary Fig. 5A–C). When dendritic arbours of Adol-DGNs (Adol-1M, Adol-2M and Adol-4M) were compared (Supplementary Fig. 5B–D), no major significant difference could be detected across ages suggesting that their development is completed within a few weeks. However, we did find a difference in the immature Adu-DGNs neurons (Adu-1M) compared with mature ones (Adu-6M), with the latter group presenting an increased number of branches in higher order branches (Fig. 5A and Supplementary Fig. 5D), a feature that was also different from mature Adol-DGNs, indicating a distinct pattern of arborisation in mature Adu-DGNs. Optogenetic inhibition had a different effect on dendritic length depending on the temporal origin and age of the DGNs (Supplementary Table 3). It did not influence the dendritic organisation of Neo-DGNs (Fig. 5B and Supplementary Fig. 5E), which is consistent with the lack of any effect on memory. Similarly, all ages of Adol-DGNs and 1-month-old Adu-DGNs dendritic lengths were not influenced by optogenetic inhibition (Fig. 5B and Supplementary Fig. 5F–I). In contrast, the complexity of the dendritic arbour of mature Adu-DGNs was decreased by the inhibition (Fig. 5B–D), suggesting that these changes are responsible for impairment in retention. To lend weight to this hypothesis, the dendritic length was correlated to the time spent (Fig. 5E) in the area previously containing the platform. We found a positive correlation between memory retention and the dendritic length: memory retention was high in rats with longer dendrites (No Light) whereas memory retention was poor in rats with shorter dendrites.

## Discussion

In the present study, we have shown that populations of DGNs of different temporal origin are sequentially involved in spatial memory and also present distinct morphological characteristics. Indeed, Adol-DGNs are involved in memory processing within a particular time window, a function taken up by Adu-DGNs at a later time point when animals become adult. In addition, Adol- and Adu-DGNs exhibit different morphologies and morphological plasticity.

Memory development is not a singular process and learning capacity emerges sequentially from the simple to the complex. The ability to learn spatial location develops later through adolescence into early adulthood. Four- to five-week-old animals use proximal cues to navigate (an independent-hippocampal strategy) and fail to search in the correct quadrant during probe trials [3, 4]. In contrast, 6 week-old animals use a hippocampal-dependent strategy to navigate thought space that requires the establishment of a cognitive map and flexible use of the learned information (spatial relational memory). It has been suggested that the maturation of spatial processing results from the maturation of the hippocampal formation and, in particular, from its spatially responsive cells [30]. Our results suggest that the emergence of navigation through space depends upon the recruitment of DGNs generated during adolescence.

Importantly and surprisingly, this role seems to be transitory since Adol-DGNs are no longer needed (for spatial memory) as animals become adult. Although this transient role was observed by the imaging and silencing of Adol-DGN, a temporal shift was observed between activated (one-month-old) and functionally recruited (two-month-old) Adol-DGNs. To reconcile these conflicting results, we propose that this shift is due to delayed development and integration of Adol-DGNs in optogenetic experiments. Indeed, in this study animals underwent two surgeries (viral injection, optic fibre implantation) and inflammation induced by the cannula [31] and/or sedation [32, 33] are known to delay neuronal development and functional integration.

We previously reported that Neo-DGNs are not recruited when animals are learning to navigate in space either using a water maze or a dry water maze [12]. Here we confirm that Neo-DGNs do not play a role in spatial navigation when animals have reached adulthood because they are not recruited by spatial learning and silencing them during training does not impair memory retrieval. More recently, using functional imaging, we have shown that Neo-DGNs are not involved in remote retrieval-induced memory reconsolidation evaluated in the water maze [12, 13]. Neo-DGNs seem to play a role in more simple functions such as discriminating between very different contexts [12].

As expected, mature Adu-DGNs were important for memory expression, a result consistent with a previous study showing that post-training ablation of 2-month-old Adu-DGNs degrades previously acquired memories [34]. Unsurprisingly, immature Adu-DGNs were not critical for memory expression, most probably because they are less spatially tuned as shown by in vivo calcium imaging [35]. However, by combining retroviral and chemogenetic approaches we have recently discovered that at the time of spatial learning (in the water maze) immature Adu-DGNs are necessary for the process of remote retrieval-induced reconsolidation [12, 13]. We speculate that immature Adu-DGNs are primed during learning and that, once mature, they influence the neural representation of the learned information (for discussion see [12, 13]).

Given the sequential involvement of Adol- and Adu-DGNs in memory processing, we propose that the adolescent wave of DGN neurons might be involved in setting up the function (organisational value) whereas adult neurogenesis may have an adaptive value. In support of this framework, Adol-DGNs (P27-35) have been shown to be important for the maturation of social behaviour [36] whereas Adu-DGNs are essential for social memory maintenance [37]. In addition, we have recently observed that the suppression of *Rnd2*, a small Rho GTPase, in DGNs born in neonates (P1) has no effect on anxiety-like behaviour whereas its deletion in Adu-DGNs exacerbates anxiety-like behaviours [38]. Based on this data we suggest that neurogenesis is a “Baldwin mechanism” that allows “ontogenetic adaptation” to a changing environment [39]. Given this framework, deleterious life events (such as chronic stress, drugs and a high-fat diet) during perinatal life and adolescence may have different outcomes and are likely to induce a vulnerable state that conditions the occurrence of disorders later in life [40–47].

In the search for the mechanisms that might explain this different role in spatial memory, we focused on the dendritic structure of DGNs because the shape of a neuron’s dendritic tree influences how synaptic information is received and integrated, which can have functional implications [48]. In the DG, it has been shown that dendritic architectures are predictive of DGN activity during spatial exploration. In particular, in rats, DGNs with high-order dendritic branching patterns are preferentially activated during spatial exploration [49].

We first identified morphological features differentiating the three mature populations: (i) Neo-DGNs have more primary dendrites, a broader branching angle and more ramifications proximal to the soma compared to the two other populations, (ii) Adol- and Adu-DGNs are indistinguishable for most of the parameters except that mature Adu-DGNs have an increased number of high order dendrites compare to the Adol-DGNs and immature Adu-DGNs. Similarly in mice, we and others found that the dendritic organisation and the synaptic input of the DGNs depend upon the ontogenetic stage of the animals [10, 50]. There are, however, some differences between species since in mice: (i) mature Emb-DGNs and not Neo-DGNs exhibit more primary dendrites, (ii) mature Neo-DGNs exhibit a longer dendritic length compared to Adol- and Adu-DGNs [10]. Not only were differences in the dendritic arborisation studied, it was also shown that Adu-DGNs and Neo-DGNs (P6 in rats) exhibit different critical periods for cell death. Whereas Adu-DGNs die within a few days after their birth [25], Neo-DGNs exhibit a delayed critical period for survival as they die between 2 and 6 months of age [11]. Furthermore, Emb-DGNs and Adol-DGNs exhibit a distinct survival dynamic from that described for Neo-DGNs [11, 51] illustrating a heterogeneity of maturation processes within cohorts of developmentally generated DGNs. Future studies will be of interest to determine whether their temporal origin determines their cellular physiology as described for early embryonic DGNs [52] and for GABAergic neurons in Ammon’s Horn [53].

Moreover, we did not observe any time-dependent evolution in the dendritic complexity of Adol-DGNs indicating that maturity is reached within four weeks. We have previously shown in mice that the dendritic arbour of Emb-DGNs and Neo-DGNs reaches maturity within 6 weeks [10]. This suggests that developmentally born neurons mature faster than Adu-DGNs which keep on growing for at least 3 months (Table S2 in [15]), and show an extended period of morphological [54, 55] and physiological [56] maturation. This heterogeneity in the rate of maturation of DGNs could be due to intrinsic differences but also to differences in their local environment [55, 57].

When we compared the effect of DGN inhibition, we found differential reactivity in mature DGNs born in the neonates, adolescent and adult animals. Developmentally-born neurons were not sensitive to light inhibition. In contrast, optogenetic inhibition of Adu-DGNs led to a reduction in dendritic complexity suggesting that these changes were responsible for impairment in memory expression. Even though we were expecting to see anatomical differences in Adol-2M DGNs that could explain the impairment in retrieval, we did not find any effect of the inhibition on the dendritic arbour. As stated previously, insults inflicted during adolescence and adulthood have different outcomes, pointing to a different level of sensitivity in these populations [14, 15]. The maturation of different neurotransmitters and neuromodulators and their signalling cascades in the hippocampus has not yet been achieved at adolescence, and it has been suggested that the maturation of glutamate receptors plays a key role in the “switch” from juvenile- to adult-like functioning [58]. For example, changes in the subunit composition of AMPA receptors have been described around P21–30 [59, 60] or it may be that mGluR5 is necessary for proper neurogenesis at P7 and P28, but is no longer necessary at later time points [61]. For this reason, we speculate that, since glutamate receptors change from birth to adulthood, they can regulate the development, reactivity and function of DGNs differently according to their temporal origin.

In conclusion, we have provided the first evidence of a causal relationship between the temporal origin of DGNs and their role in spatial memory that can provide novel insights with a view to disentangling the ways in which the heterogeneity of populations in the DG may contribute to differences in physiological and pathological states of hippocampal function.

## Supplementary information

Supplementary Information
