# Supplementary material for: The temporal origin of dentate granule neurons dictates their role in spatial memory
Source: Mol Psychiatry. 2021 Sep 15;26(12):7130–40. doi: 10.1038/s41380-021-01276-x (PMC8873024; doi:10.1038/s41380-021-01276-x)

**SUPPLEMENTARY INFORMATION**

| **Experiments** | **Delay** | **Nb of XdU-IR cells** | **Comparison of the number of XdU–IR cells (Group effect)** | **% of XdU-Zif-268IR cells** | **Comparison of activated DGNs-IR cells (Group effect)** |
| --- | --- | --- | --- | --- | --- |
| **Adol**-DGNs | 1 M | Control : 18322 ±617  Learning : 20351 ± 1384  Cued : 23040 ± 1489  Swim : 22904 ± 1688 | F_3,37_=2.61; p=0.07 | Control : 7.33 ± 1.2  Learning : 15.30 ± 1.09  Cued : 8.29 ± 0.95  Swim : 6.45 ± 0.80 | F_3,37_=16.67 ; p=0.001 |
| **Adol**-DGNs | 2 M | Control : 18948 ± 2848  Learning : 15980 ± 1257  Cued: 16140 ± 940 | F_2,18_=0.85; p=0.45 | Control : 1.70 ± 0.50  Learning : 1.41 ± 0.23  Cued: 1.37 ± 0.51 | F_2,18_=0.20, p=0.82 |
| **Adol**-DGNs | 3 M | Control : 11068 ± 1752  Learning : 11680 ± 783  Cued : 10700 ± 975 | F_2,16_=0.20; p=0.83 | Control : 2.75 ± 0.59  Learning 2.59 ± 0.55  Cued : 2.17 ± 0.47 | F _2,16_=0.32, p=0.74 |
| **Adu**-DGNs | 1 M | Control : 10013 ± 2713  Learning : 9583 ± 1212 | t_7_= 0.17; p=0.86 | Control : 3.87 ± 1.36  Learning : 3.83 ± 0.73 | t_7_= 0.03; p=0.98 |
| **Adu**-DGNs | 2 M | Control : 9360 ± 1655  Learning : 11240 ± 887 | t_6_= -1.11; p=0.31 | Control : 6.67 ± 2.79  Learning : 17.69 ± 1.50 | t_6_= -3.86; p=0.008 |
| **Adu**-DGNs | 3 M | Control : 5792 ± 1038  Learning : 5580 ± 717 | t_10_= 0.17; p=0.86 | Control : 11.75 ± 0.74  Learning : 19.28 ± 1.55 | t_10_= -3.84; p=0.003 |
| **Emb**-DGNs | 3 M | Control : 132508 ± 9219  Learning : 152655± 14733  Cued : 136854 ± 14310 | F_2,25_= 0.50; p=0.61 | Control : 5.34 ± 1.12  Learning : 6.44 ± 0.73  Cued : 5.14 ± 0.55 | F_2,25_=0.92, p=0.41 |
| **Neo**-DGNs | 10 W | Control : 19407 ± 2115  Learning : 23476 ± 1268  Cued : 21663 ± 1262 | F_2,25_=0.77; p=0.19 | Control : 8.72 ± 1.30  Learning : 8.03 ± 0.99  Cued : 7.83 ± 0.87 | F_2,25_=0.14, p=0.87 |

**Supplementary Table 1: Statistical analysis of the expression of Zif268 in DGNs.**

| **DGNs population** | **Delay** | **Number of GFP-IR cells** | **Analysis of GFP-IR cell numbers (Group effect)** | **Analysis of time spent in the quadrants (Quadrant effect)** | **Comparison of TQ to chance level** |
| --- | --- | --- | --- | --- | --- |
| **Adol**-DGNs-Arch-No-Light  **Adol**-DGNs-Arch-Light  **Adol**-DGNs-GFP-Light | 1M | 2454 ± 400  2395 ± 418  1945 ± 691 | F _2,29_=0.37, p=0.69 | F_3,39_=8.59, p<0.001 with  TQ<SE p=0.0013; TQ<NE p<0.001; TQ<SW p=0.0016.  F_3,15_=17.23, p<0.001 with  TQ< SE p<0.001; TQ<NE p<0.001; TQ<SW p<0.001  F_3,33_=10.29, p<0.001 with  TQ< SE p<0.001; TQ<NE p<0.001; TQ<SW p<0.001 | t_13_=4.07, p=0.0013  t_5_=5.35, p=0.0031  t_11_=3.89, p=0.003 |
| **Adol**-DGNs-Arch-No-Light  **Adol**-DGNs-Arch-Light  **Adol**-DGNs-GFP-Light | 2 M | 3498 ± 670  4849 ± 541  2925 ± 675 | F _2,36_=2.45, p=0.10 | F_3,36_=19.9, p<0.001 with  TQ<SE p<0.001; TQ<NE p<0.001; TQ<SW p<0.001  F _3,36_=1.84, p=0.16  F_3,36_=15.12, p<0.001 with  TQ< SE p<0.001; TQ<NE p<0.001; TQ<SW p<0.001 | t_12_=5.86, p<0.001  t_12_=1.53, p=0.15  t_12_=5.17, p<0.001 |
| **Adol**-DGNs-Arch-No-Light  **Adol**-DGNs-Arch-Light  **Adol**-DGNs-GFP-Light | 4 M | 2801 ± 634  3885 ± 457  4151 ± 617 | F _2,24_=1.55, p=0.23 | F_3,27_=5.8, p=0.003 with TQ<SE p=0.0176; TQ<NE p=0.0185; TQ<SW p=0.0024  F_3,21_=5.6, p=0.005 with TQ<SE p=0.013; TQ<NE p=0.0068; TQ<SW p=0.0068  F_3,24_=14.85, p<0.001 with  TQ< SE p<0.001; TQ<NE p<0.001; TQ<SW p<0.001 | t_9_=3.29, p=0.009  t_7_=3.40, p=0.01  t_8_=5.06, p=0.001 |
| **Adu**-DGNs-Arch-No-Light  **Adu**-DGNs-Arch-Light | 1 M | 2315 ± 581  2863 ± 320 | t_19_=0.85, p=0.41 | F_3,27_=4.477, p=0.011 with  TQ<SE p=0.019; TQ<NE p=0.016; TQ<SW p=0.010  F_3,30_=53.897, p<0.001 with  TQ< SE p<0.001; TQ<NE p<0.001; TQ<SW p<0.001 | t_9_= 2.51, p=0.033  t_10_= 10.85, p<0.001 |
| **Adu**-DGNs-Arch-No-Light  **Adu**-DGNs-Arch-Light | 6 M | 2129 ± 365  2929 ± 389 | t_21_=1.49, p=0.15 | F_3,30_=6.4, p=0.001 with  TQ<SE p=0.005; TQ<NE p=0.002; TQ<SW p=0.003  F_3,33_=2.2, p=0.1 | t_10_=3.82, p=0.003  t_11_=1.62, p=0.13 |
| **Neo-**DGNs Arch-No-Light  **Neo-**DGNs Arch—Light | 2 M | 6117 ± 2876  9126 ± 1561 | t_18_=1.01, p=0.33 | F_3,18_=12.24, p<0.001 with  TQ<SE p<0.001; TQ<NE p<0.001; TQ<SW p<0.001  F_3,36_=6.18, p=0.0017 with TQ<SE p=0.002; TQ<NE p=0.003; TQ<SW p=0.021 | t_6_=5.18, p=0.002  t_12_=3.23, p=0.0072 |

**Supplementary Table 2: Statistical analysis of behaviour performance in optogenetic experiments.**

| **DGN population** | **Delay** | **Number of rats** | **Number of DGNs analyzed** | **Sholl analysis (Light effect)** | **Ddendritic length (Light effect)** |
| --- | --- | --- | --- | --- | --- |
| **Adol**-DGNs-Arch-No-Light  **Adol**-DGNs-Arch-Light | 1 M | 6  6 | 34  36 | F_1,68_=0.1490, p=0.7007 | t_68_=0.3878, p=0.6994 |
| **Adol**-DGNs-Arch-No-Light  **Adol**-DGNs-Arch-Light | 2 M | 10  13 | 59  68 | F_1,125_=1.752, p=0.1880 | t_125_=1.325, p=0.1876 |
| **Adol**-DGNs-Arch-No-Light  **Adol**-DGNs-Arch-Light | 4 M | 8  8 | 45  42 | F_1,85_=1.807, p=0.1824 | t_85_=1.344, p=0.1824 |
| **Adu**-DGNs-Arch-No-Light  **Adu**-DGNs-Arch-Light | 1 M | 7  7 | 41  37 | F_1,76_= 0.1539, p=0.6960 | t_76_= 0.3970, p= 0.6925 |
| **Adu**-DGNs-Arch-No-Light  **Adu**-DGNs-Arch-Light | 6 M | 6  11 | 31  54 | F_1,83_=12.96, p=0.0005 | t_83_=3.603, p=0.0005 |
| **Neo-**DGNs Arch-No-Light  **Neo**-DGNs Arch-Light | 2 M | 5  5 | 32  30 | F_1,60_=0.011, p=0.9136 | t_60_=0.1084, p=0.9140 |

**Supplementary Table 3: Statistical analysis of light effect in dendritic morphology of DGNs population**


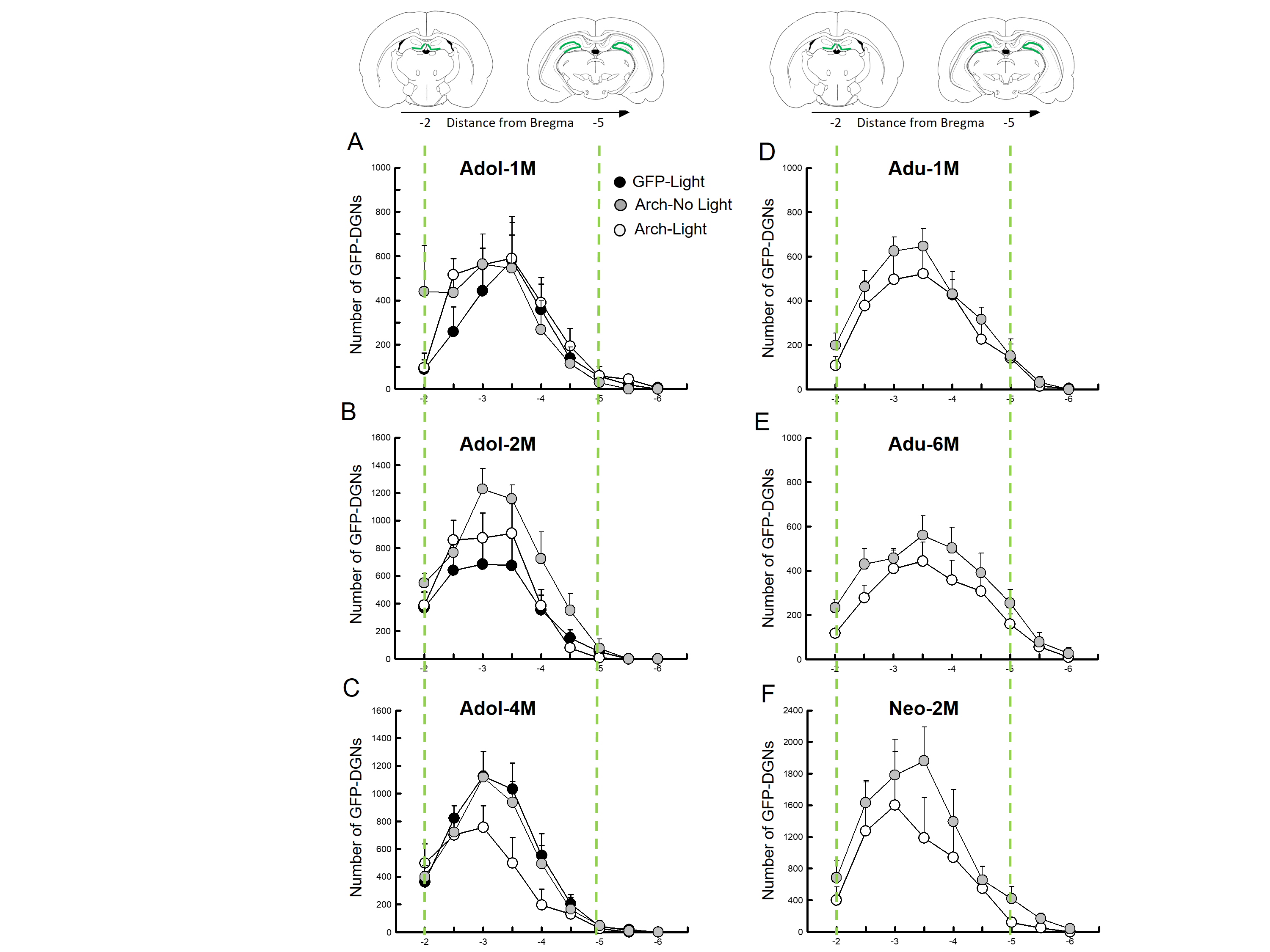


**Supplementary Fig. 1: Septotemporal distribution of Arch-eGFP or GFP DGNs.** Number of GFP-IR DGNs along the septotemporal axis of Adol-1M (**A**), Adol-2M (**B**), Adol-4M (**C**), Adu-1M (**D**), Adu-6M (**E**) and Neo-DGNs (**F**) experiments.


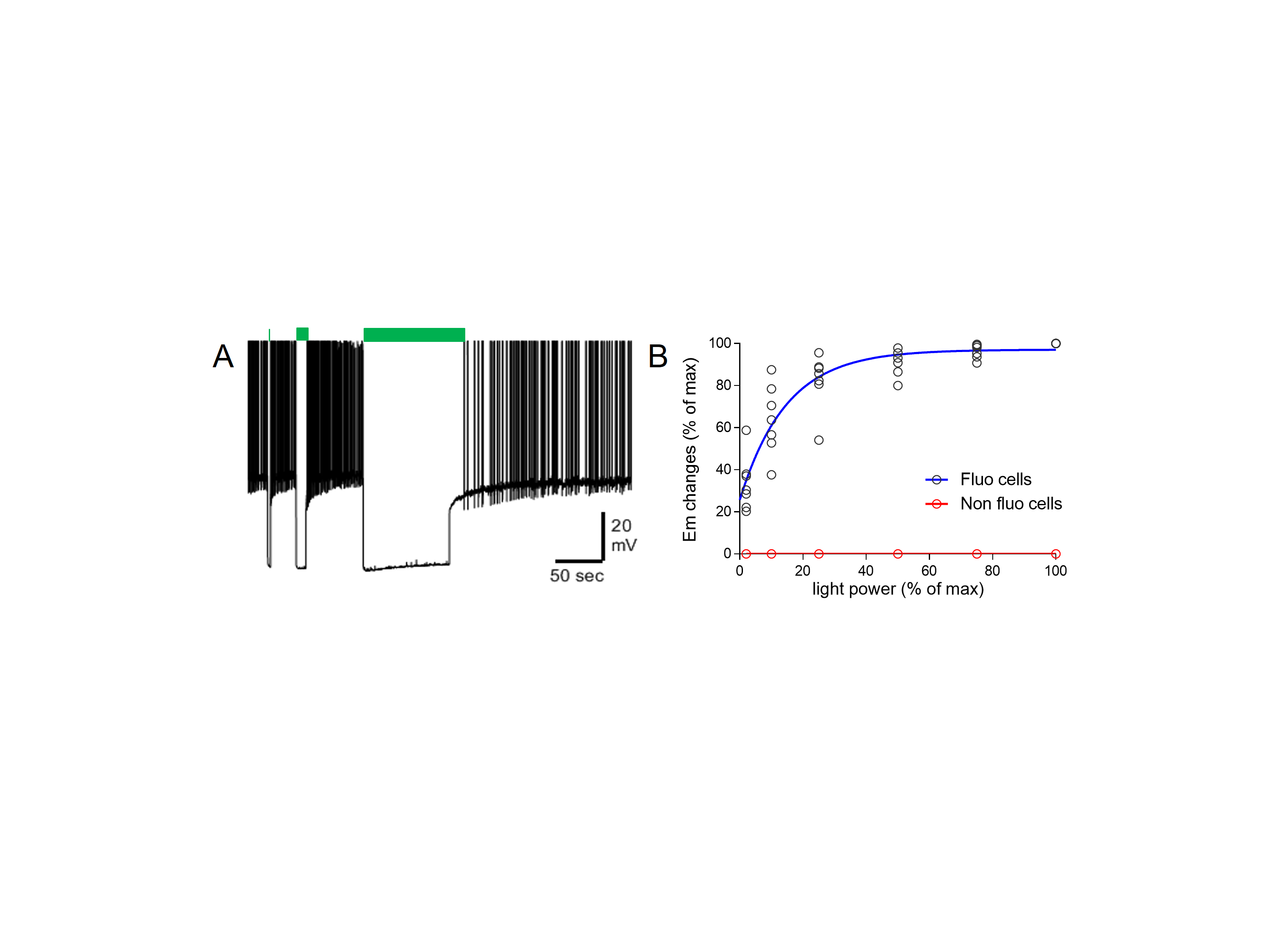


**Supplementary Fig. 2: Whole cell recordings of DGNs in rats infected with RV-Arch-eGFP.** (**A**) Representative tracing showing a whole cell patch-clamp recording under current clamp mode. Positive currents were injected in order to make the cell fire artificially. When the light was turned on (2, 10 and 90 sec, green rectangle), cell potential was instantly reduced and neurons stopped firing. (**B**) Light effect into neuronal activity, evaluated by monitoring membrane potential (Em), was power-dependent and reached a maximal effect with low power. Importantly, no effect was observed in non-fluorescent cells, in which Em was not changed.


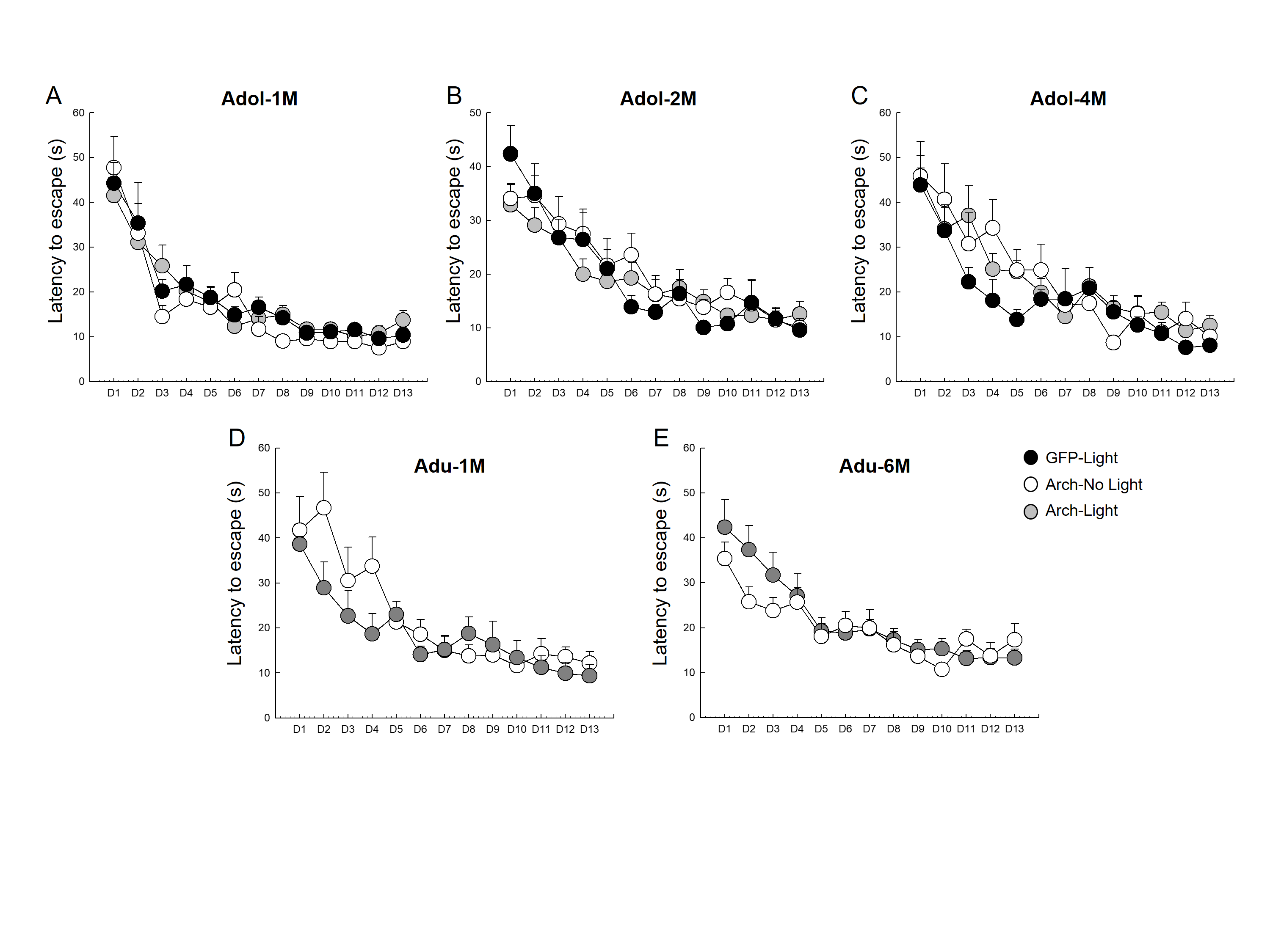


**Supplementary Fig. 3: Optical silencing of DGNs born in adolescent or adult rats does not influence acquisition of spatial memory.** RV-GFP or RV-eGFP-Arch were injected bilaterally in the DG of adolescent rats at P28 and animals were trained 1 (**A**), 2 (**B**) or 4 (**C**) months after the infection. RV-Arch-eGFP were injected bilaterally in the DG of 2-month-old rats and animals were trained 1 (**D**) or 6 (**E**) months later. Rats were trained to find a hidden platform in the water maze with half of the group trained with “Light” and the other half with “No Light”. In all groups, illumination during training did not influence learning. [Light effect: (**A**) F_2,29_=0.2964, p=0.7451; (**B**) F_2,36_=0.3257, p=0.7241; (**C**) F_2,24_=1.241, p=0.3070; (**D**) F_1,19_=0.6424, p=0.4328; (**E**) F_1,21_=0.5850, p=0.4529].


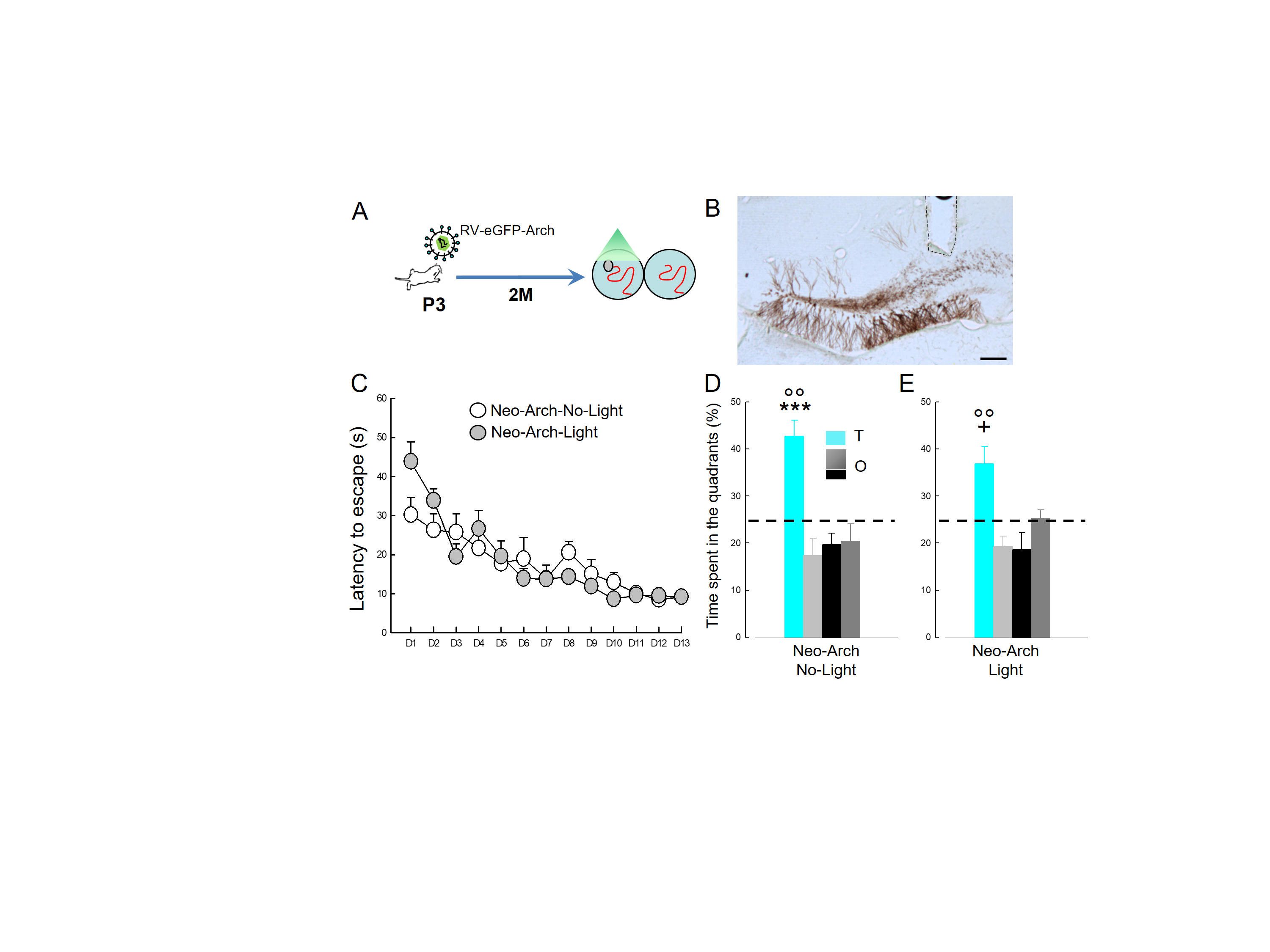


**Supplementary Fig. 4: Optical silencing of DGNs born in neonates does not influence learning or remembering.** (**A**) Experimental design: RV-Arch-eGFP were injected bilaterally in the DG of neonates at P3 (Neo). (**B**) Illustration of Neo-DGNs infected with RV-Arch-EGFP; dashed line, position where the optic fiber was placed (Bar Scale= 200µm). (**C**) Rats were trained to find a hidden platform in the water maze with half of the group trained with “Light On” and the other half with “Light Off”. Illumination during training did not influence learning (Light effect: F_1,18_=0.01421, p=0.9064). (**D, E**) Silencing 2-month-old Neo-DGNs during learning does not impaired the ability of the animals to remember the platform location. ^+^ p<0.05 compared to light grey and ***p<0.001 compared to the other quadrants. °° p<0.01 compared to chance level. T: target quadrant. O: other quadrants.

**Supplementary Fig. 5: DGNs generated during development are not affected by the light and present distinct morphological characteristics**. (**A**) Sholl distribution of the three mature DGNs population analyzed (interaction group x distance to the soma: F_78, 4095_=3.36, p<0.001) shows a different profile in Neo-DGNs. (**B**) Neo-DGNs have a higher number of primary dendrites (Group effect F_5,236_=8.258, p<0.0001) and (**C**) a broader branching angle (Group effect F_5,236_=10.35, p<0.0001) compared to Adol- and Adu-DGNs. Branching order distribution (**D**) shows different profile between the three DGNs population analyzed (interaction group x branching order : F_35,1652_=2.116, p<0.0002), of note, no differences were observed between the delays analyzed in the Adol-DGNs (Group effect F_2,135_=1.151, p=0.3194). (**E-I**) Sholl analysis shows that Neo-, Adol- nor Adu-1M DGNs are not affected by the light * p≤0.05, **p≤0.01, ***p≤0.001; + at least at p<0.05 ++ at least at p≤0.01.


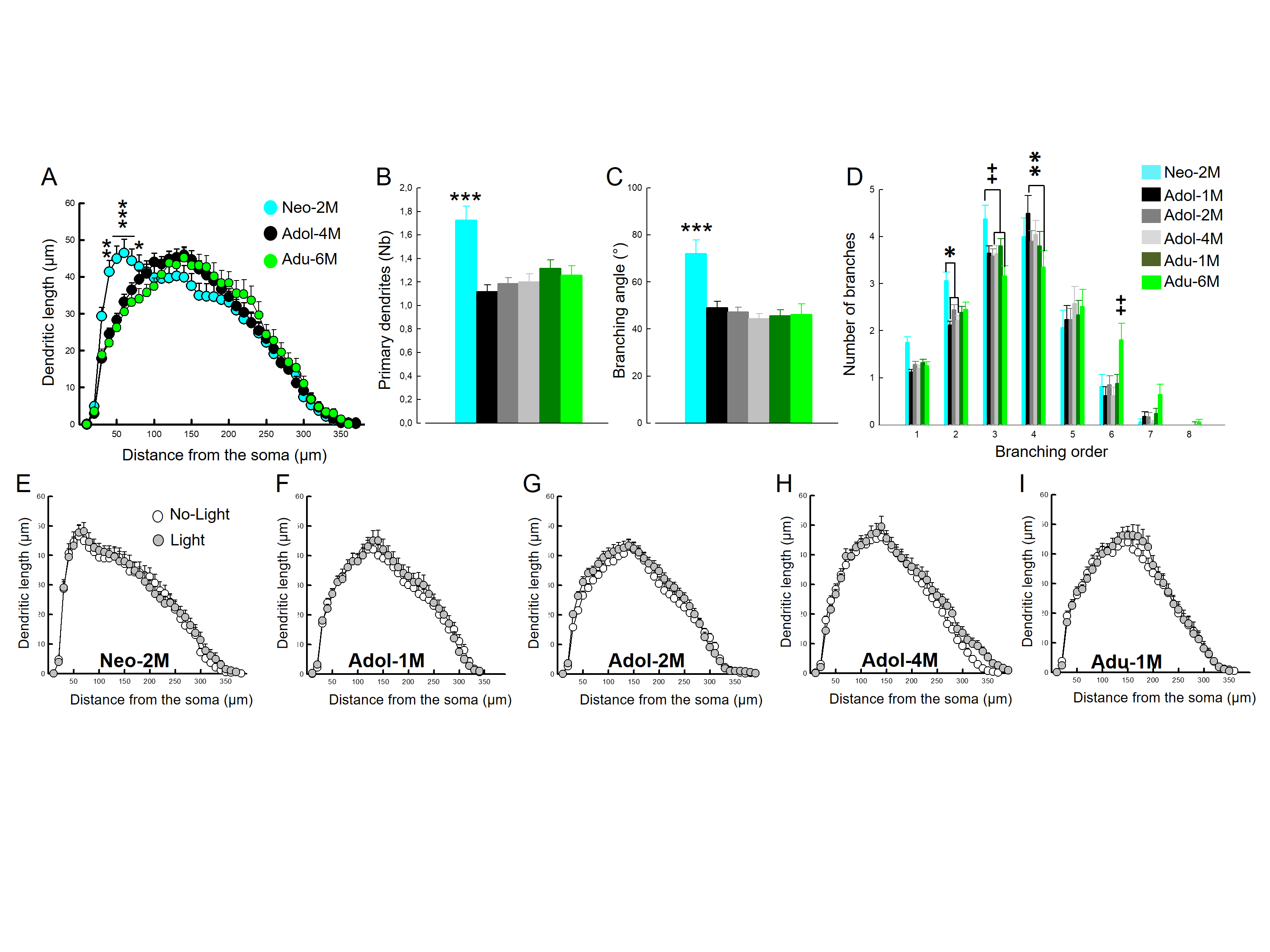

Supplement: Supplementary file 1 — Supplementary Information [file 41380_2021_1276_MOESM1_ESM.docx]
